# Metachronous bilateral renal cancer with immune checkpoint blockade-mediated eradication of bone metastasis: case report

**DOI:** 10.3389/fonc.2026.1785561

**Published:** 2026-04-01

**Authors:** Dimitar Metodiev, Tsvetan Borisov, Pierre-Alexis Da Costa, Alaeddine Redissi, Isabelle Cremer, Mila Petrova, Yann A. Vano, Lubka T. Roumenina, Dilyan Ferdinandov

**Affiliations:** 1 Clinical Pathology Laboratory, MHAT “Nadezda” Women’s Health Hospital, Sofia, Bulgaria; 2 Neuropathological Laboratory, St. Ivan Rilski University Hospital, Sofia, Bulgaria; 3 Department of General and Clinical Pathology, Faculty of Medicine, Medical University of Sofia, Sofia, Bulgaria; 4 Department of Neurosurgery, Faculty of Medicine, Medical University of Sofia, Sofia, Bulgaria; 5 Centre de Recherche des Cordeliers, Institut National de la Santé et de la Recherche Médicale, Sorbonne Université, Université de Paris Cité, Team Inflammation, Complement and Cancer, Paris, France; 6 Oncology Department, MHAT “Nadezda” Women’s Health Hospital, Sofia, Bulgaria; 7 Oncology Department, Foch Hospital, Suresnes, France; 8 Versailles St Quentin University, Paris, France; 9 University Hospital Federation (FHU) COMET, Paris, France; 10 Clinic of Neurosurgery, St Ivan Rilski University Hospital, Sofia, Bulgaria

**Keywords:** bone metastasis, immune checkpoint inhibitors, renal cell carcinoma, Sarcomatoid dedifferentiation, tertiary lymphoid structures, tumor immune microenvironment

## Abstract

**Background:**

The immune landscape of renal cell carcinoma (RCC) is a key determinant of response to immune checkpoint inhibitors (ICIs). Yet, direct comparisons of immune infiltrates across histologic subtypes and metastatic sites within the same patient are rarely possible.

**Clinical case:**

A 71-year-old man underwent left-sided nephrectomy for clear cell RCC (ccRCC) in 2013. He developed a right-sided renal and liver-capsule lesion in 2024, with an enlarged paracaval lymph node. Partial right-sided nephrectomy, liver resection, and lymphadenectomy confirmed RCC with prominent sarcomatoid histology (sRCC). Chest CT and pelvic MRI scan did not detect metastases, but the cervical spine was not explored due to lack of symptoms. Two cycles of nivolumab plus ipilimumab were performed before discovering severe cervical spine pain. MRI identified a previously missed metastasis to the C4 vertebral body. Corpectomy achieved nearly complete tumor resection supplemented with fusion. Immunohistochemistry and multiplex immunofluorescence for PD-L1/CD3/CD20/CD163/C5aR1/CD31/CA9 were performed on primary tumors and metastases.

**Results:**

Here we show (1) robust CD3^+^ and CD20^+^ infiltration in primary tumors and metastases, with immune cells in direct contact with tumor cells or organized in lymphoid aggregates/tertiary lymphoid structures; (2) PD-L1 overexpression in sRCC and nodal metastasis but absent in post-immunotherapy bone lesions; (3) increased CD163^+^ macrophages in sRCC and metastases versus ccRCC.

**Conclusions:**

This case illustrates that ICIs can induce near-complete eradication of bone metastases; that bone is not an immune-exempt organ, with massive intratumoral immune cell infiltration; and that immediate immune-mediated tumor clearance prevents structural skeletal damage, which merits careful surveillance.

## Introduction

Renal cell carcinoma (RCC) is a heterogeneous malignancy comprising distinct histological subtypes, with clear cell RCC (ccRCC) being the most common. Among these subtypes, sarcomatoid dedifferentiation is an uncommon but clinically significant feature that can arise in any RCC background and is associated with aggressive biology and poor prognosis (1). RCC tumors with sarcomatoid histology (sRCC) are characterized by high-grade transformation, early metastasis, and resistance to conventional therapies, including tyrosine kinase inhibitors (2). However, these tumors display a dense immune infiltrate and express high levels of programmed death-ligand 1 (PD-L1), features that suggest sensitivity to immune checkpoint inhibitors (ICIs) targeting PD-1/PD-L1 or CTLA-4 pathways (2–4).

Bone metastases are common in advanced RCC and, irrespective of the origin of the primary tumor, are notorious for poor response to ICIs, potentially due to the unique immunosuppressive and stromal environment of the bone marrow niche (5–9). However, emerging data from retrospective analyses and subgroup evaluations in ICIs trials and case reports indicate that bone metastases can indeed respond to checkpoint blockade (10).

Recent studies have emphasized the importance of the tumor immune microenvironment (TME) in predicting response to ICIs, particularly in histologically aggressive RCC subtypes. The spatial distribution and the density of immune cells, and the formation of tertiary lymphoid structures (TLS), have all been associated with improved outcomes under ICI therapy (11, 12). TLS are immune-organized temporary structures formed under inflammatory conditions, mainly composed of lymphocytes and dendritic cells, to support local immune response. Clinical evidence is drawn from cohort-level analyses, but only a few cases allow for a direct comparison of the immune architecture between different histological subtypes of RCC or between primary and metastatic lesions within the same patient.

Here, we report a clinically and pathologically informative case of metachronous bilateral RCC comprising an initial ccRCC, followed 11 years later by an sRCC with nodal and bony vertebral metastases. The case is notable for a rapid and near-complete histologic regression of an initially occult bone metastasis after only two cycles of combined nivolumab–ipilimumab therapy. By integrating comparative histology, immunohistochemistry, and multiplex immunofluorescence across primary and metastatic sites, this report provides a well-annotated view of immune microenvironmental remodeling associated with therapeutic response, while highlighting clinically relevant consequences of rapid immune-mediated tumor clearance in bone.

## Case presentation

We present a case of a 71-year-old man admitted to a urology clinic in early 2024 with complaints of profuse night sweats, loss of appetite and weight, low-grade fever, and right-sided lower back pain. A CT scan revealed a tumor in the upper pole of the right kidney, measuring 35/23/20 mm. There was also evidence of abdominal lymphadenopathy. Preoperative laboratory tests showed anemia (Hb <90 g/l; normal range: 135–180 g/l), elevated fibrinogen (9.1 g/l; normal range: 1.5-4.5 g/l), and high CRP (15.8 mg/dl; normal range: 0-0.6 mg/dl), elevated LDH 525 U/l (normal range: 135–225 U/l), with normal levels of neutrophils, platelets and calcium. Due to a left-sided nephrectomy performed 11 years ago, with histologically confirmed ccRCC, a decision was made to perform an organ-preserving surgical intervention – partial right-sided nephrectomy. Intraoperatively, involvement of the right liver lobe was found, necessitating an atypical liver resection. An enlarged paracaval lymph node was also removed. A pathomorphological examination revealed RCC, with predominant sarcomatoid dedifferentiation (>90% of tumor volume). Areas of necrosis were observed in about 25% of the tumor. Signs of lymphovascular invasion were noted. There was infiltration into the thickened liver capsule, with early parenchymal invasion. Intra- and peritumoral infiltrates of lymphocytes and macrophages were observed. The residual renal parenchyma exhibited signs of chronic pyelonephritis, with a marked inflammatory activation, including microabscess formation. Metastatic involvement of a paracaval lymph node was confirmed. Based on the 8th edition TNM Classification, the disease was staged as pT4N1M0 (Stage IV). According to International Metastatic RCC Database Consortium (IMDC) criteria, the patient was classified as intermediate risk at the time of treatment initiation.

According to the European Society of Medical Oncology (ESMO) and upon local regulations, immunotherapy with nivolumab 3 mg/kg q21d and ipilimumab 1 mg/kg q21d for 4 cycles started.

Two months after the resection of the right kidney tumor and after two courses of immunotherapy, the patient complained of severe pain in the cervical spine region, poorly responsive to standard analgesics. Potential causes of the acute cervical pain included early disease progression or oligoprogression due to ineffective treatment with vertebral instability, previously undetected metastatic involvement, mechanical fracture with neural compression, or, less likely, an immune-related inflammatory event affecting musculoskeletal structures. A consultation with a neurosurgeon was conducted, and an MRI was performed, revealing a malignant disseminated process in the fourth cervical vertebral body (C4). No initial head and neck MRI was performed because there were no symptoms in this area; therefore, this metastasis was not identified. A corpectomy of C4 was performed with excision of the tumor. Postoperatively, the patient was discussed for continuing the immunotherapy with the same doses. The decision was made because the bony lesion was interpreted as either oligoprogression or a preexisting lesion not detected before treatment, and because its pathomorphological examination showed an almost complete absence of viable carcinoma cells, extensive necrosis, and dense immune cell infiltration. After two more treatment cycles, the patient was admitted to the Oncology Department with fever, emesis, diarrhea, extreme fatigue, and arterial hypotension. After gastro- and colonoscopy, including biopsy control, an immune-mediated inflammatory process of the gastrointestinal tract was excluded. Due to persistence of the hypotension and low sodium level grade 1, the patient was consulted at an Endocrinology Unit, where immune-related hypocorticism was diagnosed (grade II). Treatment with methylprednisolone 1.5 mg/kg was started. PET-CT ruled out secondary involvement of the adrenal glands. After tapering off the corticosteroid to 10 mg methylprednisolone daily, immunotherapy with nivolumab 480 mg q28d was reinitiated. Hypocorticism was controlled, and corticosteroid supplementation was suspended. Twenty-two months after the diagnosis of sRCC, the patient is in stable condition, with no evidence of progression as confirmed by targeted imaging studies, and renal function remained normal after partial nephrectomy involving half of the remaining kidney.

The patient study was conducted in accordance with the ethical principles of the Declaration of Helsinki. The patient gave informed consent for this study, and the research was approved by the Ethical Committee of St. Ivan Rilski Hospital, Medical University Sofia (No7/24.10.1023).

## Histological assessment and immunohistochemistry

The materials were fixed in 10% neutral buffered formalin for 24 hours and paraffin-embedded using the Leica TP1020 Semi-enclosed Benchtop Tissue Processor (Leica Biosystems, Wetzlar, Hesse, Germany). Tissue fragments were sliced into 4-μm sections on a manual rotary microtome (Leica Biosystems) and mounted on adhesive slides by one-hour incubation at 60 °C. No decalcification was required for the bone metastasis material because the trabeculae were significantly reduced, which did not pose any difficulties during cutting. The deparaffinization was done by a heated paraffin embedding module (Cat. No. EG1150H, Leica Biosystems). The sections were routinely stained with hematoxylin and eosin. Immunohistochemical examinations were performed with the following antibodies: anti-CD4 (clone EP 204) rabbit monoclonal antibody (ready-to-use, Cell Marque, cat# 48274, Rocklin, CA, USA), anti-CD8 (clone C8/144B) mouse monoclonal antibody (ready-to-use, Cell Marque, cat# ALI 3160 G7, Rocklin, CA, USA), anti-CD20 (clone L26) mouse monoclonal antibody (ready-to-use, Zytomed Systems, cat# BMS003, Berlin, Germany), anti-CD163, rabbit polyclonal antibody (Affinity Biosciences Cat # DF8235, 1:400 dilution), anti- PD-L1 (clone 22C3), mouse monoclonal antibody (1:50 dilution; Dako, Cat# M3653, Santa Clara, CA, USA), anti-CD31 (clone EPR3095) rabbit monoclonal anti-human antibody (1:800 dilution, Abcam, Cat # ab134168), anti-C5aR1 (clone 8D6) rat monoclonal antibody (1:100 dilution, Santa Cruz Biotechnology Cat # sc-53788). The stainings for PD-L1, CD4, CD8, CD20 and CD163, CD31, and C5aR1 were performed in a research workup and were not part of the routine diagnostic practice.

## Multiplex immunofluorescence

Multiplex immunofluorescence staining was performed in a research setting on 4 µm-thick FFPE tissue sections, with a panel of 6 antibodies and the Opal 6-Plex Manual Detection Kit (NEL861001KT, Akoya Biosciences, Marlborough, Massachusetts, USA). Briefly, after deparaffinization, pretreatment was performed with Bond Epitope Retrieval Solution 2 EDTA (Leica) for 20 min.

The stainings were performed on an automated stainer (Leica BOND RX) following the manufacturer’s guidelines, then manually mounted with Prolong glass antifade mounting (Invitrogen) and scanned on the PhenoImager^®^ HT multispectral imaging platform (Akoya Biosciences, Marlborough, Massachusetts, USA) at 20x magnification.

The following antibodies were used: anti-CD3 (monoclonal mouse anti-CD3 clone: F7.2.38) for T cells, anti-CD20 (monoclonal mouse anti-CD20cy clone L26) for B cells, anti-CD21 (polyclonal rabbit anti-CD21 clone ab227662, Abcam) and anti-CD23 (monoclonal mouse anti-CD23 [SP23] ab16702) for TLS maturity, anti-PNAd (monoclonal rat anti-mouse PNAd Carbohydrate Epitope) for high endothelial venules, and anti-CA9 (anti-Carbonic anhydrase 9, Abcam ab243660)), for the tumor cells. Nuclei were stained with 10X spectral DAPI (FP1490, Akoya Biosciences, Marlborough, Massachusetts, USA). The antibodies were associated with TSA fluorophores: Opal 480, Opal 520, Opal 620, Opal 650, Opal 690, and Opal 780.

Finally, the slides were visualized and analyzed using HALO AI v.3.6 software (Indica Labs, Albuquerque, New Mexico, USA).

## Discussion

**Figure 1:**
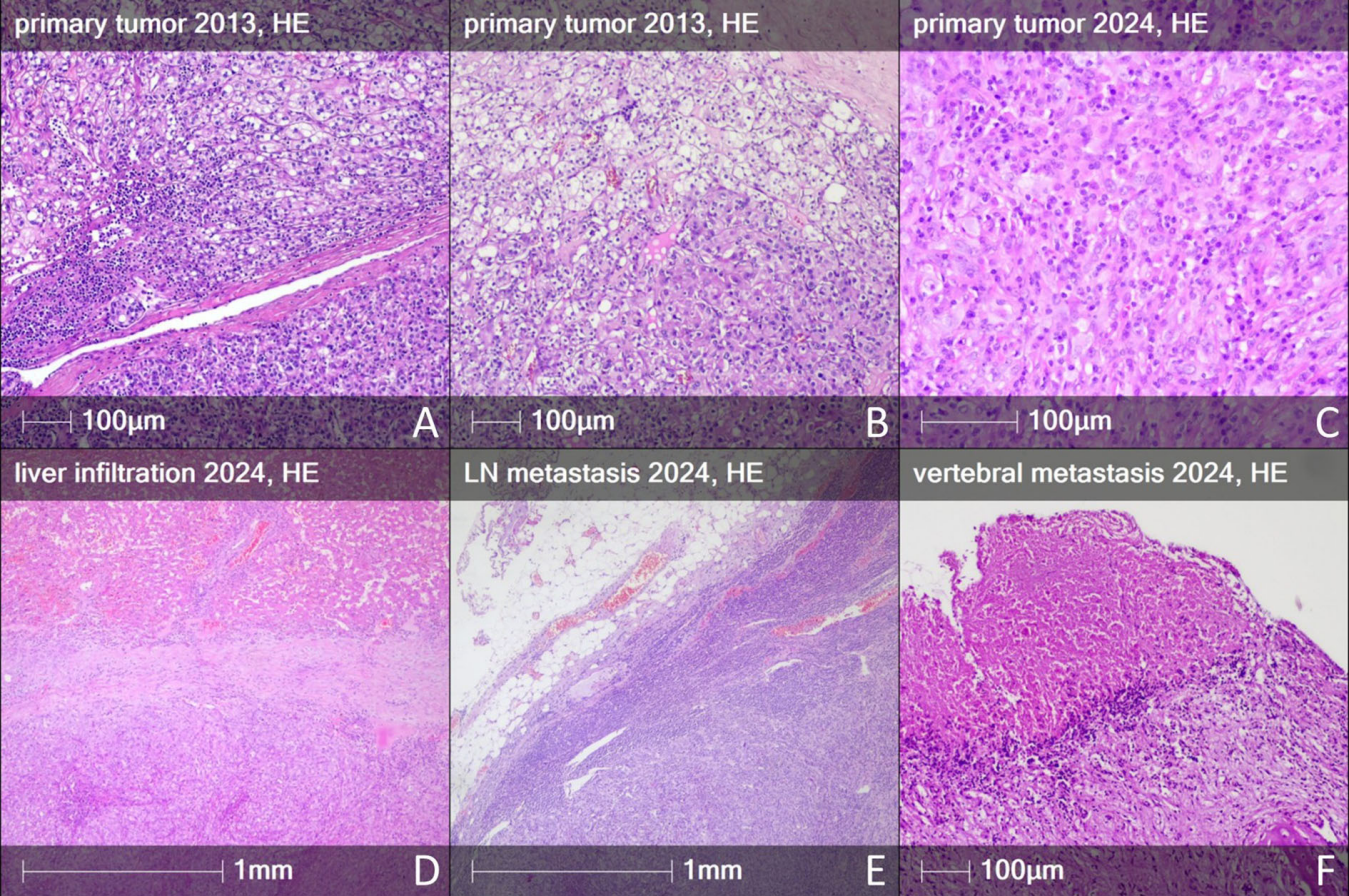
Histology of the primary tumors and metastases of the patient. **(A)** Primary tumor 2013 – RCC with conventional phenotype. **(B)** Conventional ccRCC with minimal focal sarcomatoid dedifferentiation. **(C)** Primary tumor 2024 – RCC, with sarcomatoid dedifferentiation, and abundant immune cell infiltrate. **(D)** Early liver infiltration from the sarcomatoid RCC. **(E)** Macrometastasis from sarcomatoid RCC in the paracaval lymph node. **(F)** Area of tumor necrosis in vertebral metastasis.

This patient’s clinical course highlights several rare and clinically meaningful features in the context of RCC. He developed two histologically distinct RCCs subtypes in a metachronous manner: an initial ccRCC in the left kidney, followed by a sRCC in the right kidney more than a decade later. A comparative morphological analysis was performed between the biopsies from the left nephrectomy and the tumor in the remaining right kidney. The first tumor from 2013, with a maximum diameter of 60 mm, located in the lower pole of the left kidney, exhibited morphology consistent with ccRCC, WHO/ISUP Grade 3 (**Figure 1A**). Focally, groups of cells were observed, in about 1% of the neoplastic volume, with sarcomatoid characteristics (**Figure 1B**). Therefore, the final diagnosis was ccRCC with scant sarcomatoid dedifferentiation. Tumor emboli were found in the venous vessels, within the segmental branches of the renal veins. The tumor also displayed ecstatic aberrant blood vessels, typical for ccRCC (13, 14). The tumor from 2024 in the right kidney was located in the upper pole (**Figure 1C**) and directly infiltrated the liver capsule (**Figure 1D**). The histological assessment demonstrated predominantly sarcomatoid characteristics, consistent with WHO/ISUP Grade 4. Notably, there was a dense infiltration of immune cells with peritumoral and intratumoral distribution. High microvessel density (capillary type) is observed; aberrant tumor blood vessels are absent. The metastasis in the paracaval lymph node also showed an abundant presence of tumor cells and immune infiltrate (**Figure 1E**). Pathomorphological examination of the bone metastasis specimen (**Figure 1F**) revealed an almost complete absence of carcinoma cells, large necrotic areas, and an abundant presence of mononuclear immune cells with the formation of immature tertiary lymphoid structures (TLS), such as those observed in individual areas of primary tumors of the left and right kidney.

**Figure 2:**
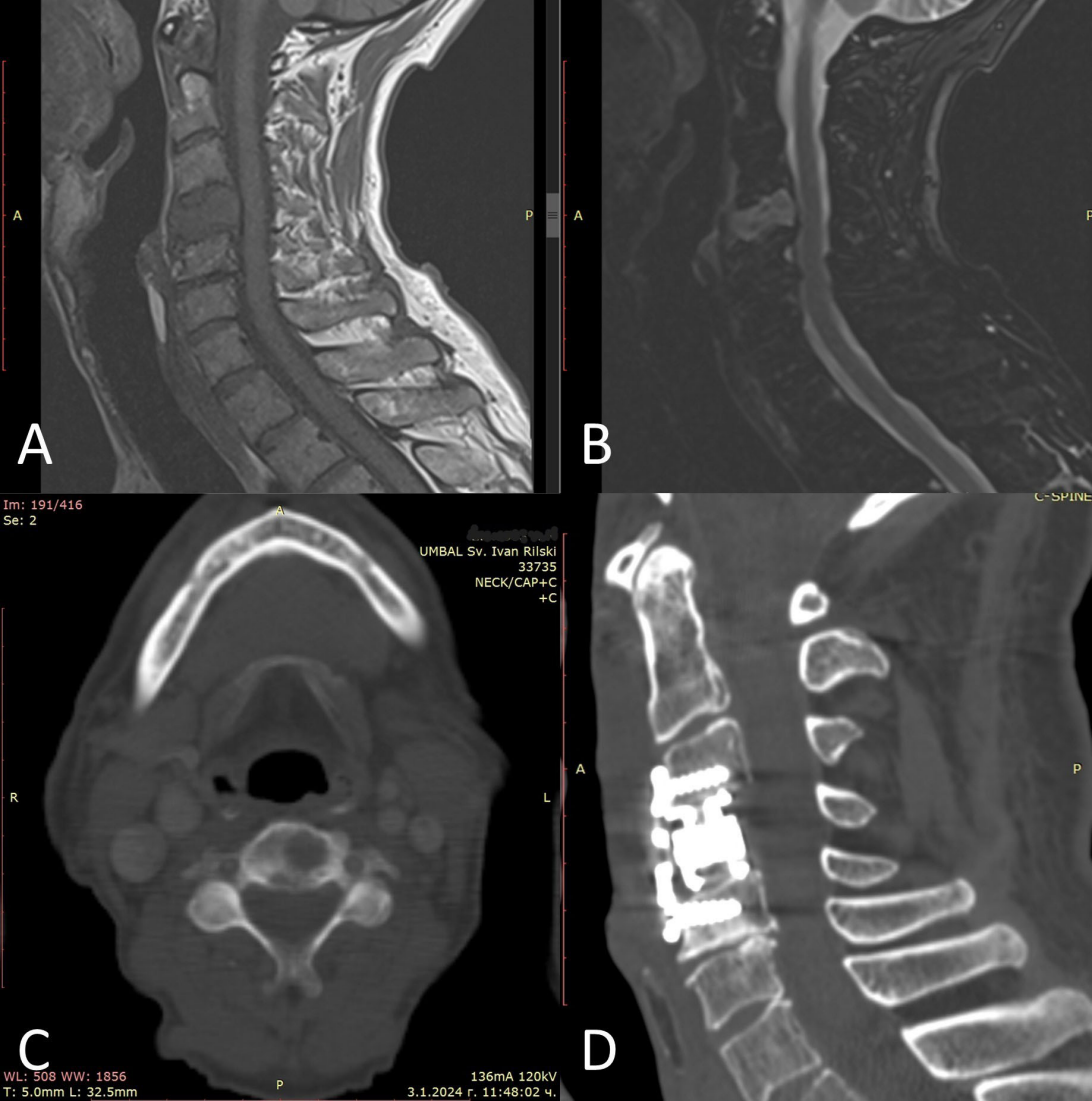
Magnetic resonance imaging of the cervical spine indicates a malignant disseminated process in the C4 vertebra, with characteristics of a metastatic lesion. **(A)** T1 sagittal sequence; **(B)** T2 TIRM (Turbo Inversion Recovery Magnitude) sagittal sequence; **(C)** Preoperative axial CT scan image demonstrating osteolytic vertebral lesions; **(D)** Postoperative sagittal CT scan image showing corpectomy and vertebral column stabilization.

While metachronous bilateral RCCs occur, they are relatively rare. Epidemiological data from large cohorts suggest that approximately 1.5% of patients with RCC develop a contralateral renal tumor more than five years after the first diagnosis, with most second tumors sharing the same histology, typically clear cell or papillary RCC (15). In contrast, the delayed appearance of a high-grade sarcomatoid carcinoma represents a highly uncommon clinical trajectory. Another important aspect of this case is the presence of vertebral metastasis (**Figures 2A–C**), which responded remarkably well to immunotherapy but caused a bone fracture, necessitating surgical intervention (**Figure 2D**).

Although there are isolated case reports of synchronous bilateral tumors with different histologic subtypes, such as combinations of clear cell and chromophobe RCC (16) or clear cell and thyroid-like RCC (17). These presentations are distinct from the case described here, where the tumors occurred 11 years apart and exhibited different biological behavior, immune profiles, and therapeutic responses. These reports also lacked longitudinal follow-up, immune profiling, or assessment of immunotherapy responsiveness.

**Figure 3:**
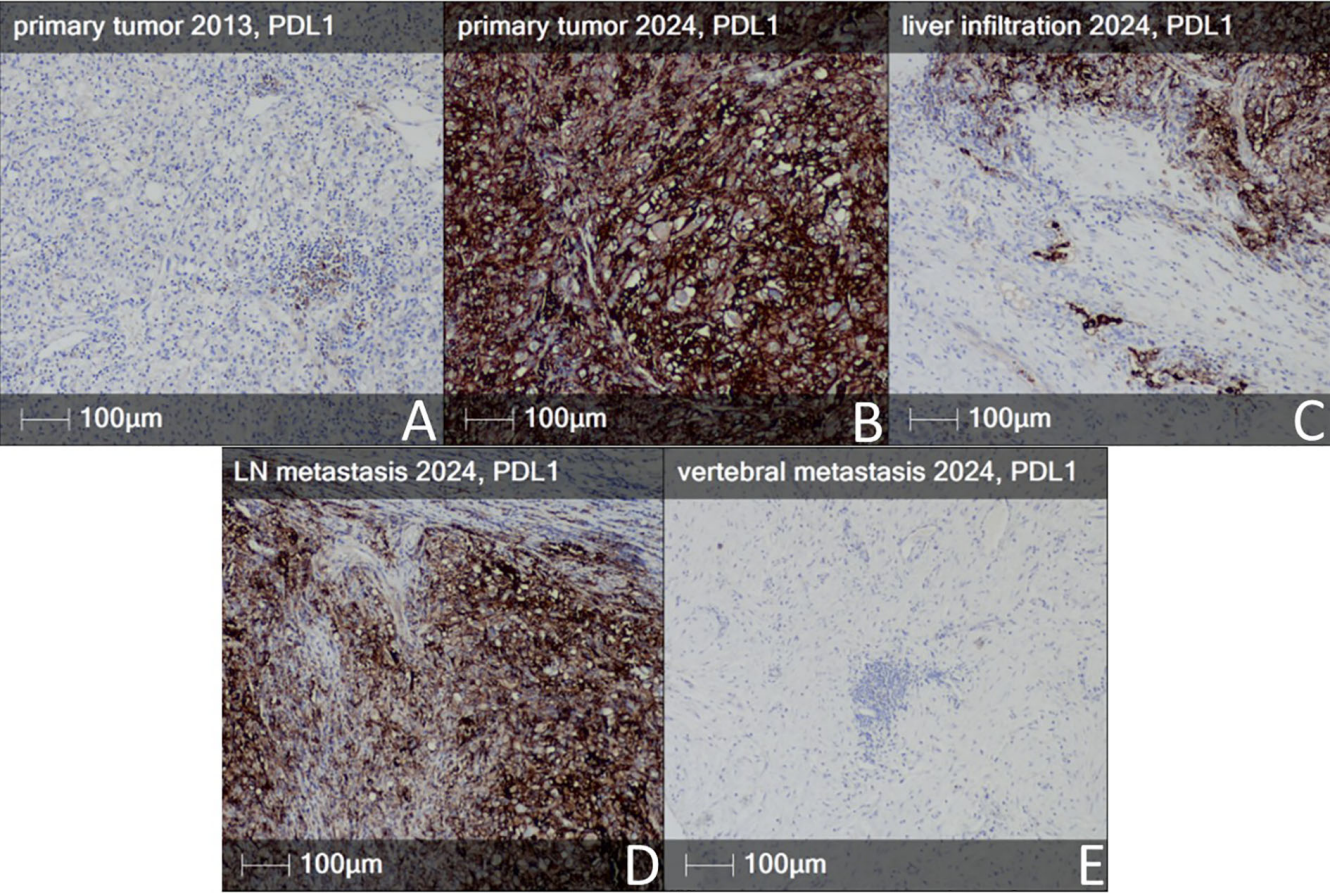
PD-L1 staining in the different tumors and metastases of the patient. **(A)** Primary tumor 2013 - mild PD-L1 expression in immune cells infiltrates. **(B)** PD-L1 overexpression in primary tumor 2024 - RCC, with sarcomatoid dedifferentiation, in neoplastic cells, as well as in immune cells. **(C)** Early liver infiltration from the sarcomatoid RCC, with PD-L1 overexpression. **(D)** Metastatic paracaval lymph node from the sarcomatoid RCC with PD-L1 overexpression. **(E)** Complete absence of PD-L1 immunoreactivity in the vertebral metastasis (IHC-PD-L1).

Sarcomatoid dedifferentiation in RCC, regardless of the underlying histologic subtype, is associated with a poor prognosis, characterized by a median survival ranging between six and thirteen months, depending on the extent of sarcomatoid features (18–21). However, sRCC is also associated with abundant immune cell infiltration and high expression of PD-L1, features that suggest sensitivity to ICIs (1, 22). In our patient, immunohistochemistry revealed strong membranous PD-L1 expression in approximately all neoplastic cells and immune infiltrate, in both the primary sRCC and the lymph node metastasis. In contrast, in the archival ccRCC specimen, including the block containing the focal sarcomatoid component, PD-L1 expression was minimal and focal. Weak PD-L1 positivity was confined to scattered immune cells within the tumor stroma, without preferential localization to the sarcomatoid focus (**Figures 3A–D**). No staining was detected in the bone metastasis, but this biopsy was taken after two courses of immunotherapy with ipilimumab and nivolumab (**Figure 3E**).

This case also allowed for intra-patient comparison of TME over time and between subtypes. The sarcomatoid tumor and its metastases exhibited higher CD163+ macrophage infiltration compared to the earlier ccRCC (**Supplementary Figure 1**), in line with prior studies showing that CD163-polarized immunosuppressive tumor-associated macrophages are enriched in aggressive and metastatic RCC (23). They also showed positivity for the complement receptor C5aR1 (**Supplementary Figure 2A**), the signaling through which contributes to the immunosuppressive tumor microenvironment (24, 25). These immunosuppressive macrophages likely contribute to tumor progression and immune evasion by promoting angiogenesis (high microvascular density of the vertebral metastasis shown at **Supplementary Figure 2B**), extracellular matrix remodeling, and suppression of cytotoxic lymphocyte function (26, 27). The marked difference in immune cell composition between the two tumors suggests significant remodeling of the TME during disease evolution (**Supplementary Table 1**).

**Figure 4:**
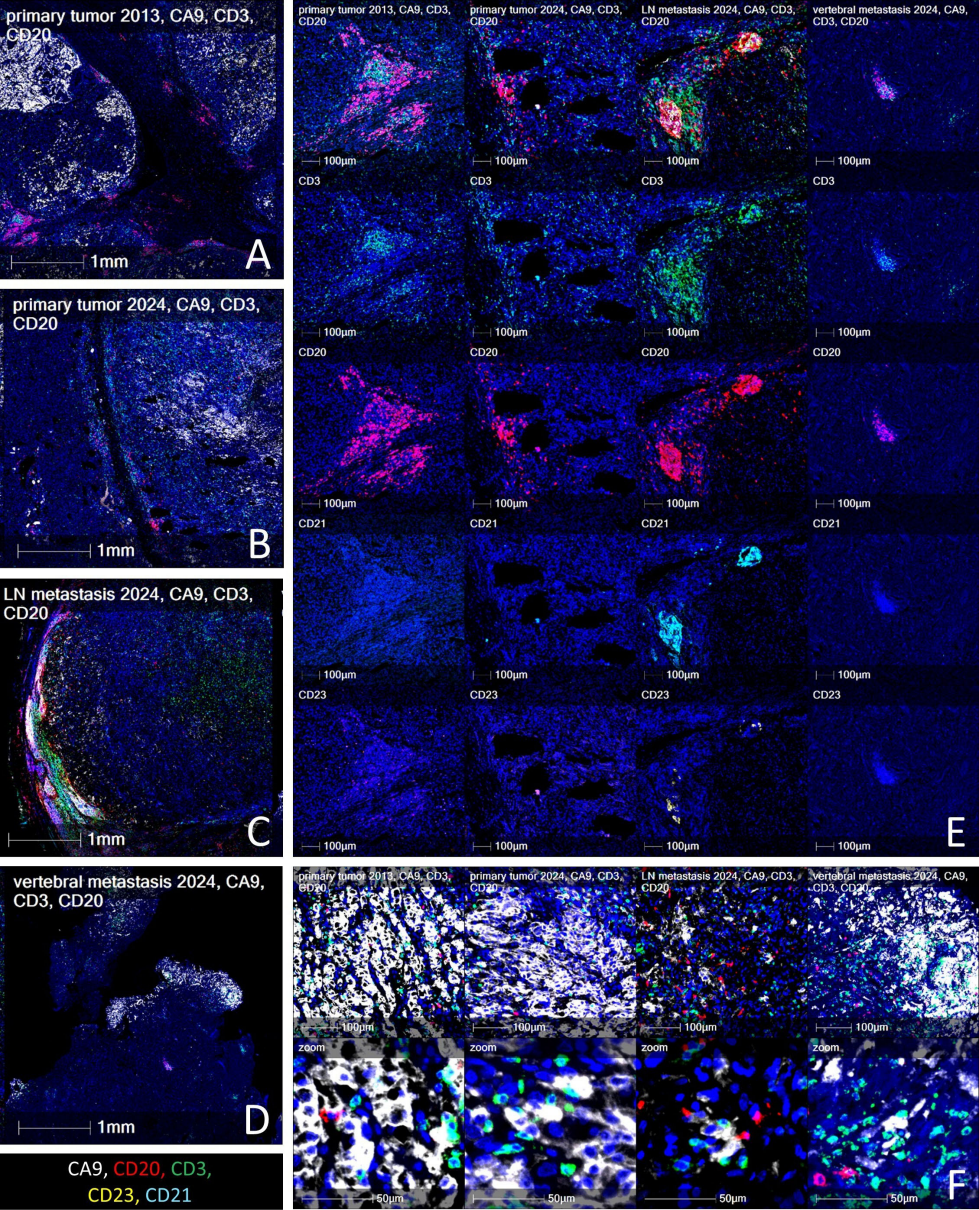
Spatial distribution of lymphocyte infiltrates in the patient’s different tumors and metastases**. (A–D)** Low-power field images illustrating the presence of tertiary lymphoid structures in the **(A)** primary ccRCC tumor from 2013. **(B)** Primary sRCC tumor from 2024. **(C)** LN metastasis from 2024. **(D)** Vertebral metastasis from 2024. **(E)** High power field illustrating the presence and maturity of tertiary lymphoid structures in the primary tumors and the two metastases. Since lymph nodes typically have organized areas of T, B, and follicular dendritic cells, this may not be a proper TLS formed under pathological conditions. **(F)** High magnification image, showing the close contact between the T and B cells with the tumor cells in the two primary tumors and the two metastases. Color code: CA9 (renal cancer) – white; CD3 (T cells) – green; B cells (CD20) – red; Follicular dendritic cells/tertiary lymphoid structures maturity markers CD21 (cyan) and CD23 (yellow).

The richness and the spatial distribution of lymphocyte infiltrates across all tumor sites are also notable (**Supplementary Figures 3**, **4**; **Figures 4A–D**). The presence of lymphoid aggregates and TLS in the bone metastasis and lymph node suggests a potential for localized, organized immune responses capable of driving tumor regression (**Figure 4E**). The primary tumors and the bone metastasis after immunotherapy presented with immature TLS (over 50 lymphocytes, positive for CD3 and CD20, but negative for CD21, CD23 and PNAd, as defined by (27) and lymphoid aggregates (T and B cell aggregates of less than 50 cells). The immature TLS, though, are not predictive for the response to immunotherapy, contrary to the mature ones (11, 28, 29). Lymph node presents what looks like a mature TLS (positive for T and B cells and CD21+/CD23+ follicular dendritic cells, and PNAd+ high endothelial venules, **Figure 4E**, **Supplementary Figure 5**). However, since lymph nodes normally have organized areas of T, B, and follicular dendritic cells, this may not be a true TLS formed under pathological conditions. Nevertheless, it is located in the tumor-infiltrated area and in direct contact with tumor cells, consistent with a tumor-associated lymphoid structure exhibiting features of mature TLS rather than a preserved native nodal follicle. It is nevertheless important to note that their anti-tumor action may be similar. Moreover, CD3+ T cells and CD20+ B cells were found in direct contact with tumor cells in the ccRCC, the sRCC, and in both metastatic lesions (**Figure 4F**), suggesting that the immune response was not limited to the peritumoral stroma but penetrated the tumor parenchyma. Moreover, the CA9-positive tumor cells in the vertebral metastasis (**Figure 4F**), surrounded by T cells, were located exclusively in necrotic areas (the necrotic zone shown in **Figure 1F** corresponds to the CA9-positive area in **Figure 1D**), illustrating the efficacy of the immunotherapy. This infiltrative pattern contrasts with immune-excluded tumors, which are typically more resistant to ICIs (30). Direct physical contact between T cells, particularly CD8^+^ cytotoxic T lymphocytes and CD4^+^ helper T cells, and tumor cells is considered a key determinant of effective anti-tumor immunity and response to immune checkpoint blockade (31). Spatial proximity facilitates T cell receptor (TCR) engagement with tumor-derived peptide–MHC complexes, enabling the release of cytotoxic granules by CD8^+^ T cells and the orchestration of immune responses by CD4^+^ T cells through cytokine production. Tumors exhibiting a so-called “inflamed” or “immune-infiltrated” phenotype, in which T cells are not excluded from but are embedded within tumor nests, are more likely to respond to PD-1/PD-L1 blockade (32). Conversely, immune-excluded or immune-desert tumors tend to resist such therapies, possibly due to stromal or vascular barriers that prevent T cell access. Thus, the presence of intratumoral T cells in direct contact with cancer cells, as observed across all lesions in our case, including a regressing bone metastasis, suggests a functionally engaged immune microenvironment and provides mechanistic support for the observed clinical response to dual checkpoint blockade. Indeed, the histopathological examination of the C4 vertebral body following corpectomy showed few viable tumor cells but abundant mononuclear immune infiltrates, with T and B cells forming lymphoid aggregates. This indicates that a substantial immune response, likely driven by nivolumab plus ipilimumab, had eradicated the tumor parenchyma within two cycles of therapy. Such a rapid and complete response of bone metastases to immune checkpoint blockade is rarely reported. Although bone is frequently considered an immunologically challenging site with poor responsiveness to immunotherapy (33), this case suggests that sRCC metastases in bone can be highly responsive to dual immune checkpoint blockade.

Neoadjuvant immunotherapy in RCC is still investigational, but has preliminarily demonstrated the safety and efficacy of neoadjuvant low-dose lenvatinib plus pembrolizumab followed by a radical nephrectomy in ccRCC patients at high-risk of recurrence or progression (34). Moreover, pathologic response and post-treatment influx of CD8+/CD39+ cells associate with prolonged disease-free survival following neoadjuvant avelumab plus axitinib therapy, and the patients with a major pathological response had a distinct spatial co-localization gene signatures of tumor and immune cells in the tumor microenvironment (35), similarly to the pattern we see in our patient. Importantly, this case supports the potential of neoadjuvant immunotherapy in RCC, as it demonstrates that even occult metastatic lesions can undergo rapid and complete histologic regression after exposure to dual ICIs. It suggests that preoperative immunotherapy may help eradicate micrometastatic disease and improve surgical outcomes in aggressive RCC subtypes.

This histological eradication of bone metastasis also underscores the need to reconsider how response to immunotherapy is assessed. Imaging may not immediately reflect pathological response, and radiologically stable or persistent lesions could, in fact, be devoid of tumor cells, particularly in osseous tissues, where residual cavities or highly vascularized fibrotic areas may be misinterpreted. Furthermore, rapid immune-mediated tumor clearance within the bone can pose structural risks. As seen in this patient, the area once occupied by the tumor became structurally compromised, necessitating surgical stabilization. In cases of highly responsive metastatic sRCC treated with dual checkpoint blockade, orthopedic risk must be anticipated and managed proactively. This clinical scenario also emphasizes the value of reporting sarcomatoid components in RCC pathology to guide therapeutic decision-making. It calls into question the notion that bone metastases are intrinsically resistant to immunotherapy and suggests that even occult lesions can respond robustly when the lesions have a responsive immune infiltrate and the treatment is appropriately tailored. At the same time, the structural consequences of rapid tumor clearance in bone should not be underestimated, and closer multidisciplinary collaboration is warranted to anticipate and manage such risks.

## Conclusions

This case offers a unique insight into the natural history and immunotherapy responsiveness of RCC subtypes. It underscores the importance of combining detailed pathological assessment with immune profiling, highlighting the need for prospective studies. As immunotherapies continue to reshape treatment paradigms in RCC, individual cases such as this contribute valuable information for future clinical and translational strategies.

The ICIs can induce near-complete eradication of bone metastases; that bone is not an immune-exempt organ, with massive intratumoral immune cell infiltration; and that immediate immune-mediated tumor clearance prevents structural skeletal damage, which merits careful surveillance.

## Data Availability

The original contributions presented in the study are included in the article/**Supplementary Material**. Further inquiries can be directed to the corresponding author.

## Supplementary material

Supplementary Figure 1 Immunosupressive myeloid infiltration of the different tumors and metastases of the patient. **(A)** Primary tumor 2013 - low immunoreactivity for CD163 marker. **(B–E)** CD163-positive macrophages are highly represented in **(B)** primary sarcomatoid RCC, **(C)** its liver infiltration and its metastases in the **(D)** Lymph Node and the **(E)** C4 vertebral region.

Supplementary Figure 2 **(A)** C5aR1 staining in the vertebral metastasis. **(B)** CD31 vascular staining in the vertebral metastasis.

Supplementary Figure 3 Lymphoid infiltrate. **(A)** CD4 T cells, **(B)** CD8 T cells, and **(C)** B cell (CD20+) infiltration of the different tumors and metastases of the patient.

Supplementary Figure 4 Lymphocyte infiltrate in the sRCC tumor from 2024 in the kidney and in the liver. **(A)** Low power field representing the primary sRCC tumor at the interface between the kidney and the liver. CA9 staining for renal cancer cells (white). Autofluorescence of the hepatocytes (brown). T cells detected by CD3 staining (green) and B cells, detected by CD20 staining (red). **(B, C)** Intratumor heterogeneity for the immune infiltrate, higher power fields. **(B)** Intermediate-low infiltration with T cells. **(C)** Strong infiltration with T and B cells. **(D)** Example of the liver, infiltrated with tumor cells, accompanied by T and B cell infiltrate.

Supplementary Figure 5 Characterization of the tumor-associated lymphoid structure with TLS features in the lymph node metastasis from 2024. Images illustrate the presence of lymphoid structures (B cells, CD20+ in red and T cells, CD3+ in green), within the lymph node metastasis (renal tumor cells, CA9+ in white), showing mature phenotype, positive for high endothelial venules (PNAd+) and follicular dendritic cells/tertiary lymphoid structures maturity markers CD21 (cyan) and CD23 (yellow).

Supplementary Table 1 Semi-quantitative scores of immune markers across tumor sites reflect relative immune cell density assessed across representative high-power fields. “Low” indicates sparse infiltrates, “Moderate” indicates readily identifiable immune populations without confluence, and “High” indicates dense or confluent immune infiltration. *PD-L1 expression was semi-quantitatively categorized as absent (<1%), focal/low (1–5%), moderate (5–10%), or high (>10%). Using this approach, the archival ccRCC specimen showed focal/low PD-L1 expression confined to scattered immune cells. In contrast, the sarcomatoid RCC and nodal metastasis demonstrated high PD-L1 expression in neoplastic cells and in immune infiltrates. PD-L1 expression was absent in the vertebral metastasis following immune checkpoint blockade. **IC = immune cells. ***- = Lymph node presents what looks like a mature TLS (positive for T and B cells and follicular dendritic cells. However, since lymph nodes typically have organized areas of T, B, and follicular dendritic cells, this may not be a proper TLS formed under pathological conditions.
