# Supplementary figures and images for: Metachronous bilateral renal cancer with immune checkpoint blockade-mediated eradication of bone metastasis: case report
Source: Front Oncol. 2026 Apr 1;16:1785561. doi: 10.3389/fonc.2026.1785561 (PMC13078976; doi:10.3389/fonc.2026.1785561)

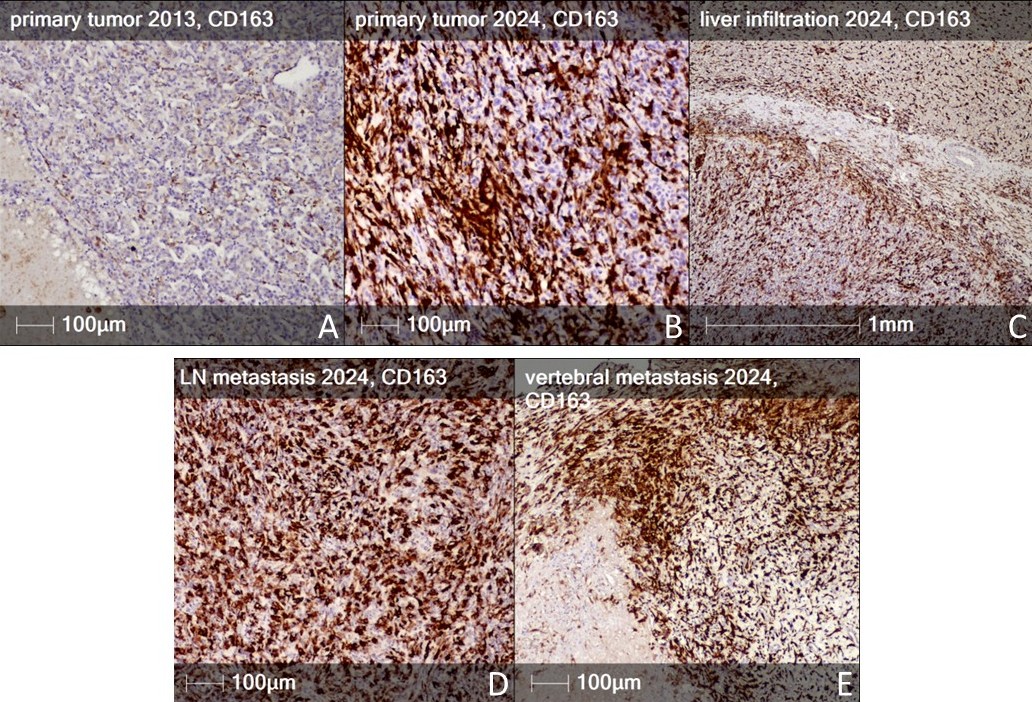

Supplement: Supplementary Figure 1 — Immunosupressive myeloid infiltration of the different tumors and metastases of the patient. (A) Primary tumor 2013 - low immunoreactivity for CD163 marker. (B–E) CD163-positive macrophages are highly represented in (B) primary sarcomatoid RCC, (C) its liver infiltration and its metastases in the (D) Lymph Node and the (E) C4 vertebral region. [file Image1.jpeg]

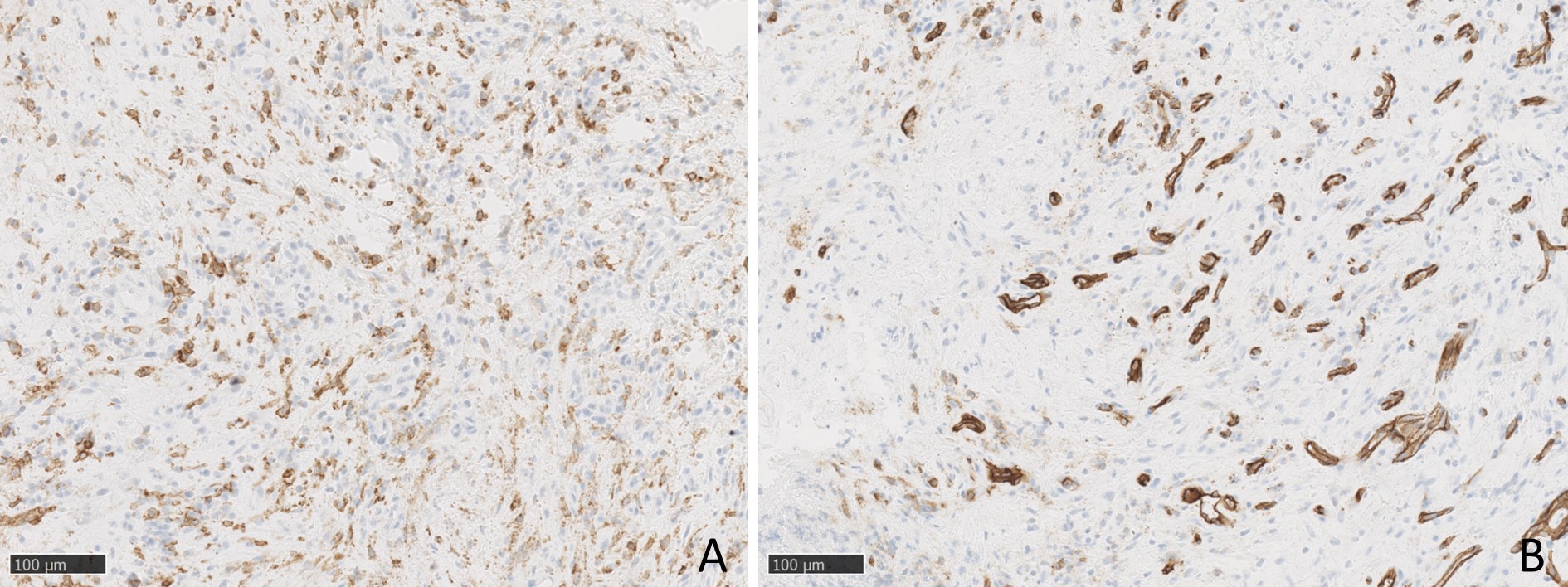

Supplement: Supplementary Figure 2 — (A) C5aR1 staining in the vertebral metastasis. (B) CD31 vascular staining in the vertebral metastasis. [file Image2.jpeg]

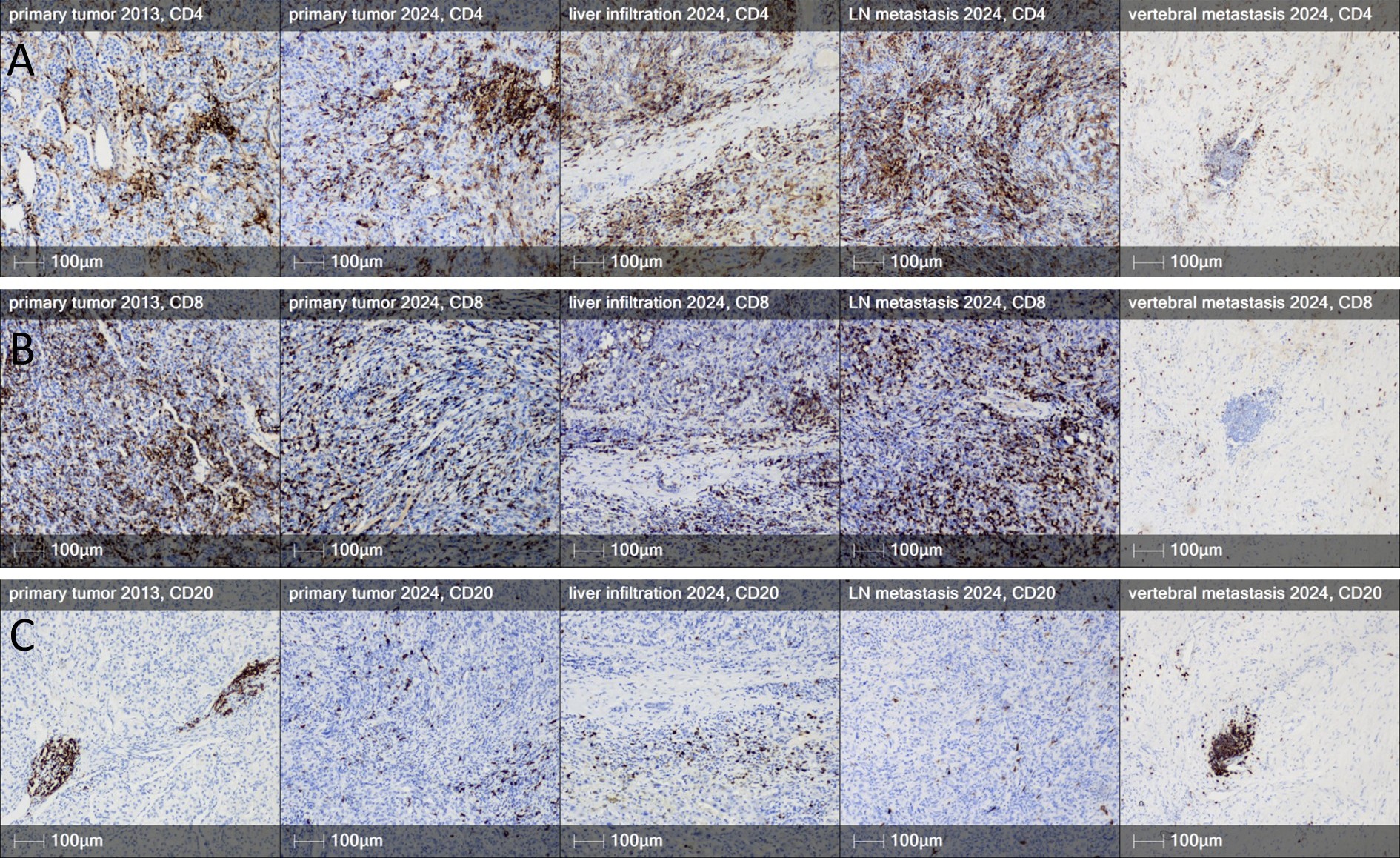

Supplement: Supplementary Figure 3 — Lymphoid infiltrate. (A) CD4 T cells, (B) CD8 T cells, and (C) B cell (CD20+) infiltration of the different tumors and metastases of the patient. [file Image3.jpeg]

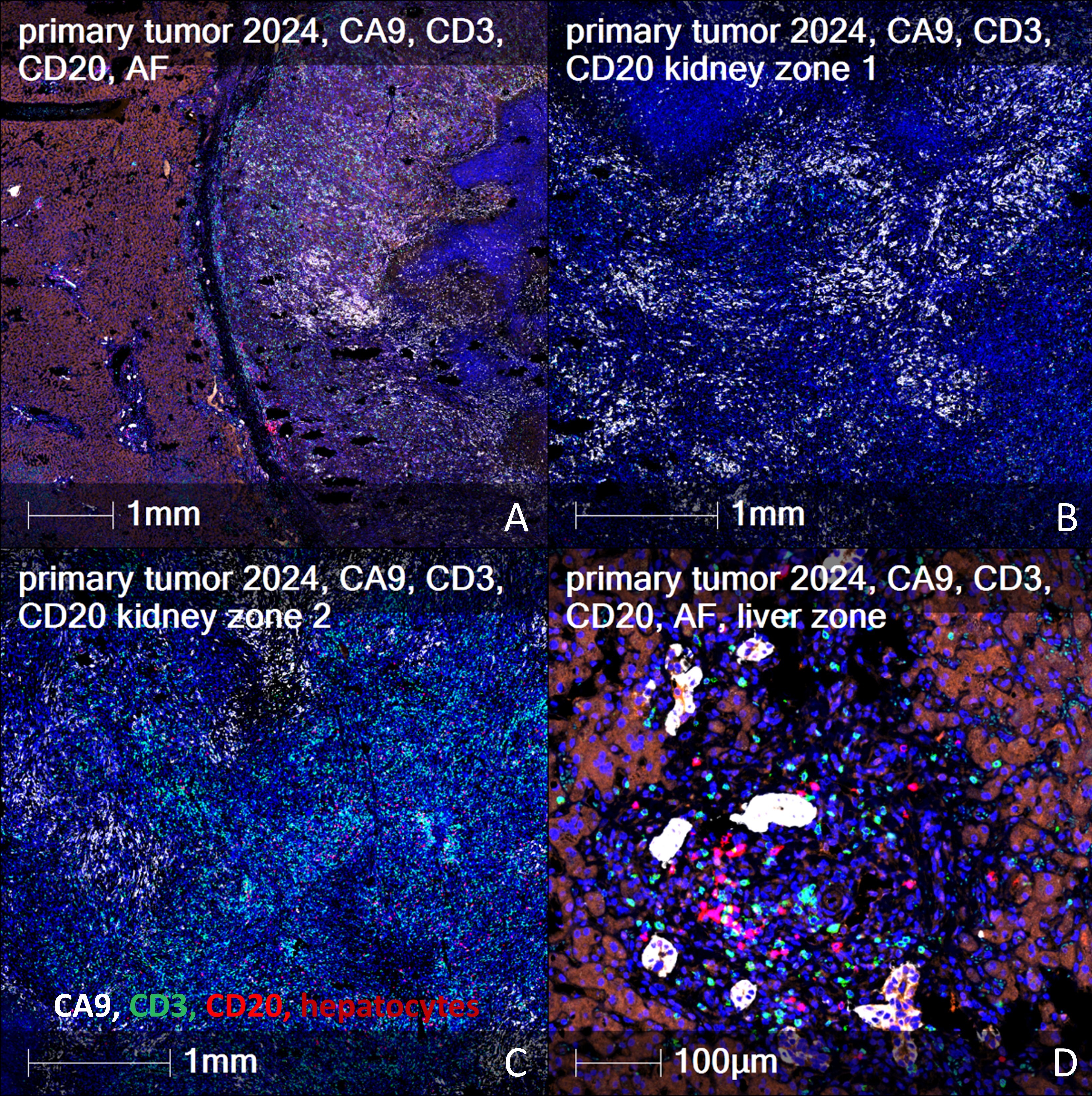

Supplement: Supplementary Figure 4 — Lymphocyte infiltrate in the sRCC tumor from 2024 in the kidney and in the liver. (A) Low power field representing the primary sRCC tumor at the interface between the kidney and the liver. CA9 staining for renal cancer cells (white). Autofluorescence of the hepatocytes (brown). T cells detected by CD3 staining (green) and B cells, detected by CD20 staining (red). (B, C) Intratumor heterogeneity for the immune infiltrate, higher power fields. (B) Intermediate-low infiltration with T cells. (C) Strong infiltration with T and B cells. (D) Example of the liver, infiltrated with tumor cells, accompanied by T and B cell infiltrate. [file Image4.jpeg]

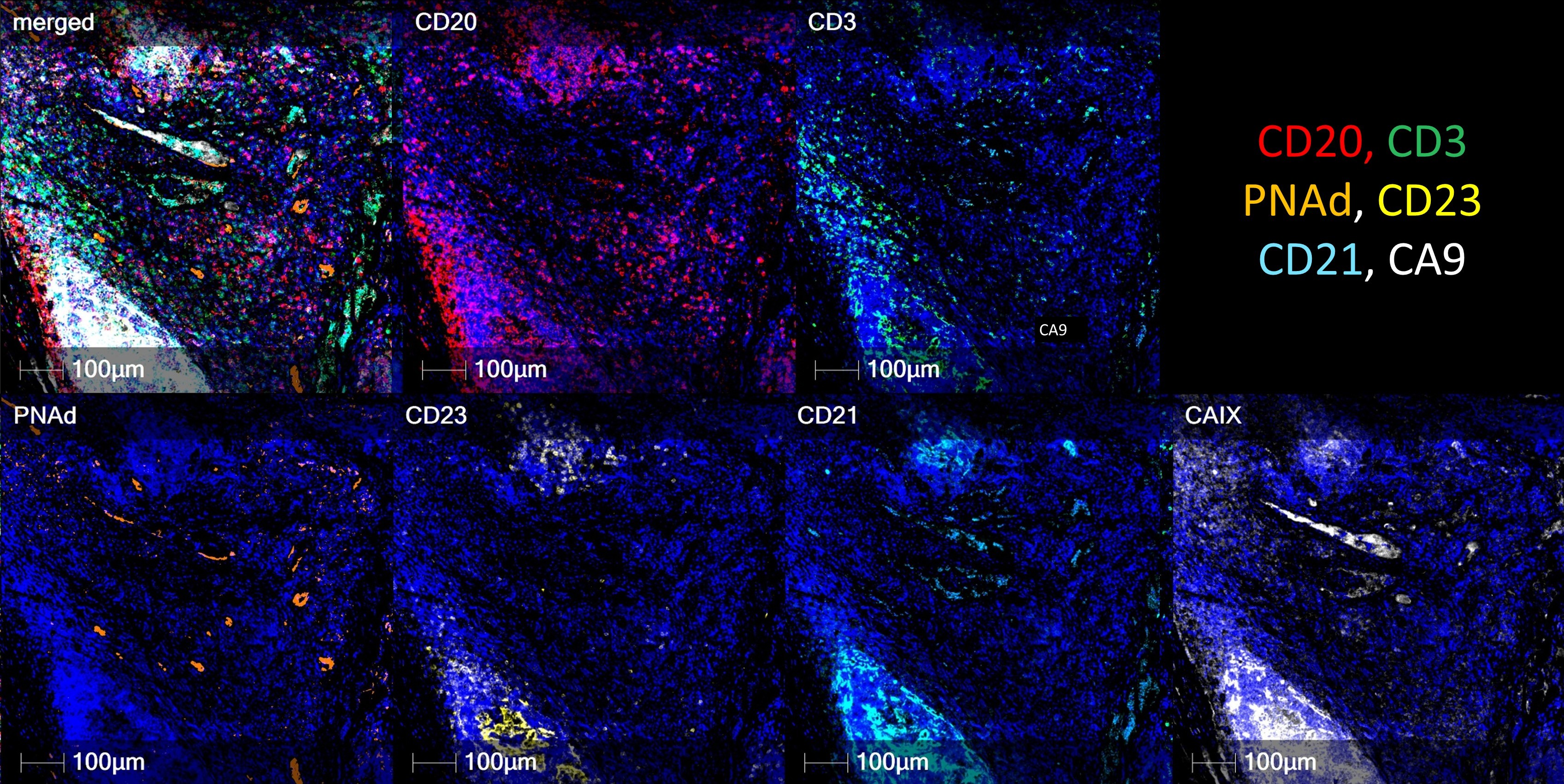

Supplement: Supplementary Figure 5 — Characterization of the tumor-associated lymphoid structure with TLS features in the lymph node metastasis from 2024. Images illustrate the presence of lymphoid structures (B cells, CD20+ in red and T cells, CD3+ in green), within the lymph node metastasis (renal tumor cells, CA9+ in white), showing mature phenotype, positive for high endothelial venules (PNAd+) and follicular dendritic cells/tertiary lymphoid structures maturity markers CD21 (cyan) and CD23 (yellow). [file Image5.jpeg]
