# Supplementary material for: Metachronous bilateral renal cancer with immune checkpoint blockade-mediated eradication of bone metastasis: case report
Source: Front Oncol. 2026 Apr 1;16:1785561. doi: 10.3389/fonc.2026.1785561 (PMC13078976; doi:10.3389/fonc.2026.1785561)
Supplement: Supplementary Table 1 — Semi-quantitative scores of immune markers across tumor sites reflect relative immune cell density assessed across representative high-power fields. “Low” indicates sparse infiltrates, “Moderate” indicates readily identifiable immune populations without confluence, and “High” indicates dense or confluent immune infiltration. *PD-L1 expression was semi-quantitatively categorized as absent (<1%), focal/low (1–5%), moderate (5–10%), or high (>10%). Using this approach, the archival ccRCC specimen showed focal/low PD-L1 expression confined to scattered immune cells. In contrast, the sarcomatoid RCC and nodal metastasis demonstrated high PD-L1 expression in neoplastic cells and in immune infiltrates. PD-L1 expression was absent in the vertebral metastasis following immune checkpoint blockade. **IC = immune cells. ***- = Lymph node presents what looks like a mature TLS (positive for T and B cells and follicular dendritic cells. However, since lymph nodes typically have organized areas of T, B, and follicular dendritic cells, this may not be a proper TLS formed under pathological conditions. [file Table1.docx]

| **Marker** | **ccRCCC from 2013** | **sRCC from 2024** | **Lymph node metastasis** | **Bone metastasis** |
| --- | --- | --- | --- | --- |
| PD-L1* | Low (IC** only) | High | High | Absent |
| CD3⁺ T cells | Low | High | -*** | High |
| CD8⁺ T cells | Low-Moderate | Moderate | - | High |
| CD20⁺ B cells | Low | Moderate | - | High |
| CD163⁺ macrophages | Low | High | High (in metastatic nests) | Moderate |
| TLS | Rare | Present | - | Present |

**Supplementary Table 1.** Semi-quantitative scores of immune markers across tumor sites reflect relative immune cell density assessed across representative high-power fields. “Low” indicates sparse infiltrates, “Moderate” indicates readily identifiable immune populations without confluence, and “High” indicates dense or confluent immune infiltration. *PD-L1 expression was semi-quantitatively categorized as absent (<1%), focal/low (1–5%), moderate (5–10%), or high (>10%). Using this approach, the archival ccRCC specimen showed focal/low PD-L1 expression confined to scattered immune cells. In contrast, the sarcomatoid RCC and nodal metastasis demonstrated high PD-L1 expression in neoplastic cells and in immune infiltrates. PD-L1 expression was absent in the vertebral metastasis following immune checkpoint blockade. **IC = immune cells. ***- = Lymph node presents what looks like a mature TLS (positive for T and B cells and follicular dendritic cells. However, since lymph nodes typically have organized areas of T, B, and follicular dendritic cells, this may not be a proper TLS formed under pathological conditions.
