# Supplementary material for: Metachronous bilateral renal cancer with immune checkpoint blockade-mediated eradication of bone metastasis: case report
Source: Front Oncol. 2026 Apr 1;16:1785561. doi: 10.3389/fonc.2026.1785561 (PMC13078976; doi:10.3389/fonc.2026.1785561)
Supplement: Supplementary file 7 [file DataSheet1.pdf]

Supplementary Figure S6. Clinical timeline of tumor evolution and immune checkpoint blockade

2013	Left radical nephrectomy<br>Clear cell RCC (WHO/ISUP G3)<br>Focal sarcomatoid dedifferentiation (<1%)
2013–2024	Disease-free interval (11 years)
Early 2024	Right renal mass + paracaval lymph node<br>Partial nephrectomy + liver capsular resection + lymphadenectomy<br>Predominantly sarcomatoid RCC (>90%)<br>pT4N1 – IMDC intermediate risk
2024	Initiation of nivolumab + ipilimumab
After 2 cycles	Acute cervical spine pain<br>MRI: C4 vertebral metastasis
2024	C4 corpectomy + spinal stabilization<br>Histology: near-complete tumor necrosis<br>Dense immune infiltrate
Follow-up	Immune-related hypocortisolism (temporary)<br>Immunotherapy resumed<br>Alive with stable disease at 22 months

This supplementary figure provides a schematic overview of the clinical course, including tumor evolution, surgical interventions, immune checkpoint inhibitor therapy, and histopathologic assessment.
